# Supplementary material for: First insight into the viral community of the cnidarian model metaorganism Aiptasia using RNA-Seq data
Source: PeerJ. 2018 Mar 1;6:e4449. doi: 10.7717/peerj.4449 (PMC5835348; doi:10.7717/peerj.4449)
Supplement: Fig. S1 [file peerj-06-4449-s005.pdf]

Raw sequence reads
297 million reads

After Quality Control, Read trimming, Adapter removal
264 million reads (88.89%)

Trimmomatic (Bolger et al. 2014), fastq-mcf (Aronesty 2011)

Removal of Aiptasia host, algal endosymbiont, PhiX, 28S rRNA sequences
8.6 million reads (2.89%)

BBsplit from BBmap (Bushnell 2016)

Paired-end read pairs based taxonomic classification
38,090 annotated sequences (bacteria, archaea, viruses)

CLARK (Ounit 2015)

Virus community: taxonomic composition and structure
3,293 sequences,
116 distinct viral taxa of 40 families

MetagenomeSeq (Paulson et al. 2013) & R package vegan (Oksanen et al. 2017)

Aiptasia *Symbiodinium* Bacteria & Archaea Virus
